# S04-6: INTEGRATE-PA-Pol: A Tool for Assessing Cross-Level Collaboration in HEPA Policies

**DOI:** 10.1093/eurpub/ckae114.216

**Published:** 2024-09-26

**Authors:** Eugen Resendiz, Deborah Salvo, Andrea Ramírez-Varela, Juliana Mejía-Grueso, Jane Moon, Michael Pratt, Josef Mitáš, Ross C Brownson

**Affiliations:** Department of Kinesiology and Health Education, The University of Texas at Austin, Austin, USA; Department of Kinesiology and Health Education, The University of Texas at Austin, Austin, USA; Department of Epidemiology, School of Public Health, UTHealth Science Center at Houston, Houston, USA; Center for Pediatric Population Health, School of Public Health, UTHealth Science Center at Houston, Houston, USA; Department of Pediatrics at McGovern Medical School, UTHealth Science Center at Houston, Houston, USA; Global Observatory for Physical Activity – GoPA!; Herbert Wertheim School of Public Health and Human Longevity Science, University of California San Diego, La Jolla, CA, USA; Herbert Wertheim School of Public Health and Human Longevity Science, University of California San Diego, La Jolla, CA, USA; Faculty of Physical Culture, Palacký University Olomouc, Olomouc, Czech Republic; Prevention Research Center, Brown School, Washington University in St. Louis, United States

## Abstract

**Purpose:**

The Physical Activity Policy at the National and City Levels Project aimed to develop and test a tool to assess how national and subnational (city and region) governments collaborate to develop and implement health-enhancing physical activity (HEPA) policies.

**Project Description:**

The development of the “Interaction between National and Local Government Levels in Development and Implementation of Physical Activity Policies Tool” (INTEGRATE-PA-Pol) took place in three phases: 1) scoping review to identify local government physical activity promotion policies and instruments for assessing them, 2) questionnaires development, and 3) cognitive response testing (validity testing and item modification) with local physical activity experts. The final tool comprises six questionnaires assessing how national and subnational governments (city and region) collaborate to develop and implement HEPA policies. An observational cross-sectional study was conducted in four Latin American countries and eight of their cities to test the feasibility of the tool used for global surveillance. GoPA! Country Contacts from each country were invited to serve as their country’s principal investigators. Country Contacts identified the main sectors, key actors, and policymakers from each sector involved in HEPA policy development and implementation at the national level, the capital city, and an additional city for participant recruitment. Between August 2022 and February 2023, online snowball sampling surveys collected data on national and subnational government informants' perspectives on the development and implementation stages of national and local HEPA policies. The pilot study demonstrated the feasibility of collecting national and subnational HEPA policy data across countries with the active collaboration of GoPA! Country Contacts. We also successfully identified governmental interactions throughout the PA policy process, suggesting suboptimal engagement between national and subnational levels.

**Conclusions:**

This tool can assist in better understanding how government levels and sectors collaborate during the HEPA policy process and will help uncover policy gaps and improve policy translation between national and subnational (city and region) governments. Our tool will contribute to developing and implementing a global public policy monitoring system to enable benchmarking and priority setting for HEPA policies.

